# Population Aging at Cross-Roads: Diverging Secular Trends in Average Cognitive Functioning and Physical Health in the Older Population of Germany

**DOI:** 10.1371/journal.pone.0136583

**Published:** 2015-08-31

**Authors:** Nadia Steiber

**Affiliations:** 1 Wittgenstein Centre (IIASA, VID/ÖAW, WU), International Institute for Applied Systems Analysis (IIASA), Laxenburg, Austria; 2 Department of Economic Sociology, University of Vienna, Vienna, Austria; University of Brescia, ITALY

## Abstract

This paper uses individual-level data from the German Socio-Economic Panel to model trends in population health in terms of cognition, physical fitness, and mental health between 2006 and 2012. The focus is on the population aged 50–90. We use a repeated population-based cross-sectional design. As outcome measures, we use SF-12 measures of physical and mental health and the Symbol-Digit Test (SDT) that captures cognitive processing speed. In line with previous research we find a highly significant *Flynn effect* on cognition; i.e., SDT scores are higher among those who were tested more recently (at the same age). This result holds for men and women, all age groups, and across all levels of education. While we observe a secular improvement in terms of cognitive functioning, at the same time, average physical and mental health has declined. The decline in average physical health is shown to be stronger for men than for women and found to be strongest for low-educated, young-old men aged 50–64: the decline over the 6-year interval in average physical health is estimated to amount to about 0.37 SD, whereas average fluid cognition improved by about 0.29 SD. This pattern of results at the population-level (trends in average population health) stands in interesting contrast to the positive association of physical health and cognitive functioning at the individual-level. The findings underscore the multi-dimensionality of health and the aging process.

## Introduction

Life expectancy has been increasing in the last decades mainly owing to a reduction in old age mortality. In this context, prior research has suggested that members of more recently born cohorts do not only tend to live longer, they also tend to enjoy better functioning and health until higher ages [1–5]. In other words, age-related decline associated with the process of ‘normal aging’ [6] has been postponed to higher ages [7]. A steadily increasing number of studies suggest that more recently born cohorts show improvements over older cohorts in physical measures of disability and are able to manage their activities of daily living until higher ages than were previous cohorts [2,3,8–11]. Moreover, prior research has consistently shown better cognitive functioning at ages 50+ in more recent cohorts [2,12–21]. Such findings of secular improvements in cognitive functioning are in line with studies showing that later generations face a lower risk of dementia [22]. Overall, it thus appears that those of advanced chronological age today show a set of characteristics typical for younger ages in older cohorts [23]. In other words, later generations show fewer signs of age-related decline in functioning and health compared to earlier generations.

Notwithstanding the cumulating evidence of increasingly healthy aging, a number of studies paint a less optimistic picture of health trends in aging societies. First, more mixed findings tend to be reported with regard to disease prevalence: in some countries members of more recent cohorts are more likely to report chronic diseases [24]. Second, while trends in milder forms of disability tend to be more favorable, in some countries rates of severe disability have increased in more recent cohorts [25,26].

Irrespective of the direction of trends, a survey of extant empirical research creates the impression that the different dimensions of age-related decline move in the same direction. This construes the notion that there is likely to be a ‘common cause explanation’ for the postponement of physical and cognitive aging [27]. However, the available evidence on the association of change in cognition with change in physical functioning and health is in fact scant, both at the individual-level and the population-level (for a review of the literature, see [28,29]). While there is consistent evidence that physical and cognitive health tend to co-vary (positive correlation at the individual-level), fairly little is known to date about the longitudinal association of individuals’ cognitive abilities with their physical functioning and health [28] (for a systematic review of research). Even less is known about the conjoint development of different dimensions of *population health*. The only study available that has investigated simultaneous cohort effects on the physical *and* cognitive capabilities of a population [2] shows that the Danish cohort of nonagenarians born in 1915 attains significantly higher scores on standardized tests of cognition (e.g., animal naming task, immediate and delayed word recall) as well as on the activities-of-daily-living scale (ADL) than the 1905 cohort. This suggests that improvements in average cognitive functioning across cohorts tend to come along with improvements in average physical capability (as measured by the ADL scale). The only other study we are aware of that has investigated trends in the average physical *and* cognitive functioning of a country’s older population [9], uses a measure of self-rated memory not a memory test as a measure of cognitive functioning. It finds significant cohort improvements between 1988 and 2004 both in abilities to carry out instrumental activities of daily living (IADL) and memory function in the Finnish population aged 65–69.

The present study contributes to this literature by investigating trends in the average cognitive functioning, physical and mental health of the older German population (ages 50–90). It uses data from two survey waves of the German Socio-Economic Panel spanning a 6-year interval. The aim is to ascertain if the three dimensions of health do in fact move in the same direction. The well-established evidence of positive associations between physical and cognitive health at the level of individuals may–based on the assumption of a common cause for age-related changes in multiple domains–lead to the expectation that improvements in average physical health come along with improvements in average cognitive functioning (conjoint trends in population health). Yet, this assumption may rest on false inference from micro-level to macro-level associations. Even if there is a positive correlation between (changes in) physical and cognitive functioning at the individual-level, there can in theory be negative correlations at the aggregate-level [30].

## Data and Methods

This study uses anonymized secondary data, collected by the German Institute for Economic Research (DIW). The German Socio-Economic Panel (SOEP) is approved as being in accordance with the standards of the Federal Republic of Germany for lawful data protection, all participants gave free and informed consent to participate in the survey. The survey ethics are monitored by an independent advisory board at the DIW. The authors signed a contract with the data holders to permit the use and publishing of data for scientific purposes.

The SOEP is a large representative survey of private households that provides, among a wealth of other information, representative health scores for the German population. A 12-Item Short-Form Health Survey (SF-12) that is considered to provide a quasi-objective measure of health [31,32] is included in the core questionnaire every other year since 2002. Cognitive testing has been carried out in 2006 and 2012 on *random sub-samples* of SOEP participants. The study utilizes these two survey waves. It includes all SOEP participants, aged 50–90 at the time of interview, who provide valid scores for all SF-12 health indicators and the cognitive test (see below for details about the outcome measures and the sample composition).

### Measures of physical and mental health

The SF-12 module in the SOEP is modelled on the classic SF-12v2 Health Survey [33] that uses 12 questions to measure functional health and well-being. The SF-12 is deemed a highly reliable and valid measure of physical and mental health in population health surveys. It has been developed by public health experts aiming for a single and continuous measure of health that is comprehensive and that minimizes measurement error in self-reported health. It covers eight health domains: *physical functioning (PF)*, *role limitations due to physical health problems (RP)*, *bodily pain (BP)*, *general health perceptions (GH)*, *energy and vitality (VT)*, *social functioning (SF)*, *role limitations due to mental health or emotional problems (RE)*, *and mental health (MH)*. Based on factor analytic analyses [34] (for detail), these domains are summarized in two superordinate dimensions: *physical health (PCS) and mental health (MCS)*. Whereas the component summary scale for physical health (PCS) focuses on functional limitations in terms of mobility and task performance (e.g., ascending stairs, lifting objects, strong physical pain, role limitations), the component summary scale for mental health (MCS) has been shown to be a valid measure of mental health and a useful screening tool for depressive disorders in general population studies [35,36]. It is based on six questions related to psychological well-being, mental balance, emotional problems, social functioning, and vitality. The exact survey questions that refer to a period of four weeks before the interview are presented in the S1 File. PCS and MCS are orthogonal by construction (i.e., the scores do not correlate). The PCS and MCS scores are z-standardized to a mean value of 50 and a SD of 10. They are norm-based allowing for a comparison of subgroups and survey waves against the 2004 norm.

While the SF-12 measure may be subject to some of the well-known limitations of self-rated health, it has a number of merits. First, it defines health less narrowly than physical health measures such as the diagnosis of specific diseases by certified doctors. Second, in contrast to medical diagnoses the SF-12 is less affected by changing health infrastructures and health knowledge (disease rates may appear to rise although in fact only the rate at which they are diagnosed rises). Third, the external validity of the measures is confirmed by a large set of studies. Fleishman and Lawrence [37], for example, report associations of the SF-12 with a set of diagnosed clinical conditions such as diabetes, asthma, high blood pressure, emphysema, a stroke, and other heart conditions. Moreover, it has been shown that declines in PCS scores are associated with significant declines in the risk of death [38,39]. The validity of the SF-12 as a measure of *population health* is also confirmed by studies that show a strong correlation between average PCS scores and the age-adjusted mortality rate at the community-level as well as a strong correlation between average MCS scores and the homicide rate at the community-level [40]. Finally, PCS scores correlate with hand-grip strength [41] (see also findings of this study), a measure that has been identified as a good predictor of health and mortality [42,43].

### Measure of cognitive functioning

The SOEP provides test scores on a Symbol-Digit Test (SDT) that is based on the Symbol-Digit-Modality-Test [44]. The SDT is a test of perceptual speed, commonly used to measure fluid intelligence [45]. Respondents sit in front of a laptop. Before the test starts, a screen image shows a series of nine graphical symbols that are assigned a number between 1 and 9. The image remains visible during the test, i.e., participants do not need to memorize the symbol-digit association. The test starts with the appearance of one of the symbols, asking respondents to match it with the correct digit as quickly as possible. It ends automatically after 90 seconds. The number of correctly assigned numbers provides a measure of respondents’ information-processing speed [45,33]. For better comparability with the SF-12 measures, we standardize the SDT score through z-transformation and subsequently transform it linearly to a mean value of 50 and a SD of 10.

The SDT is in principle designed for self-administration. In exceptional circumstances, respondents could ask the interviewer to enter the digits they suggested. This option was only available in 2006 when it was used by 17% of respondents [46,47]. To deal with potential issues of score comparability and sample selectivity related to test modalities, we carry out sensitivity analyses using two different strategies, (1) excluding test participants giving oral responses in both survey waves and (2) including test participants giving oral responses by way of imputing the score they would have obtained in the self-completion mode. Comparing the results based on the first strategy with those based on the second, we find that the conclusions from the results are robust to the strategy chosen (S2 File for details).

### Analytic strategy

**Fig 1:**
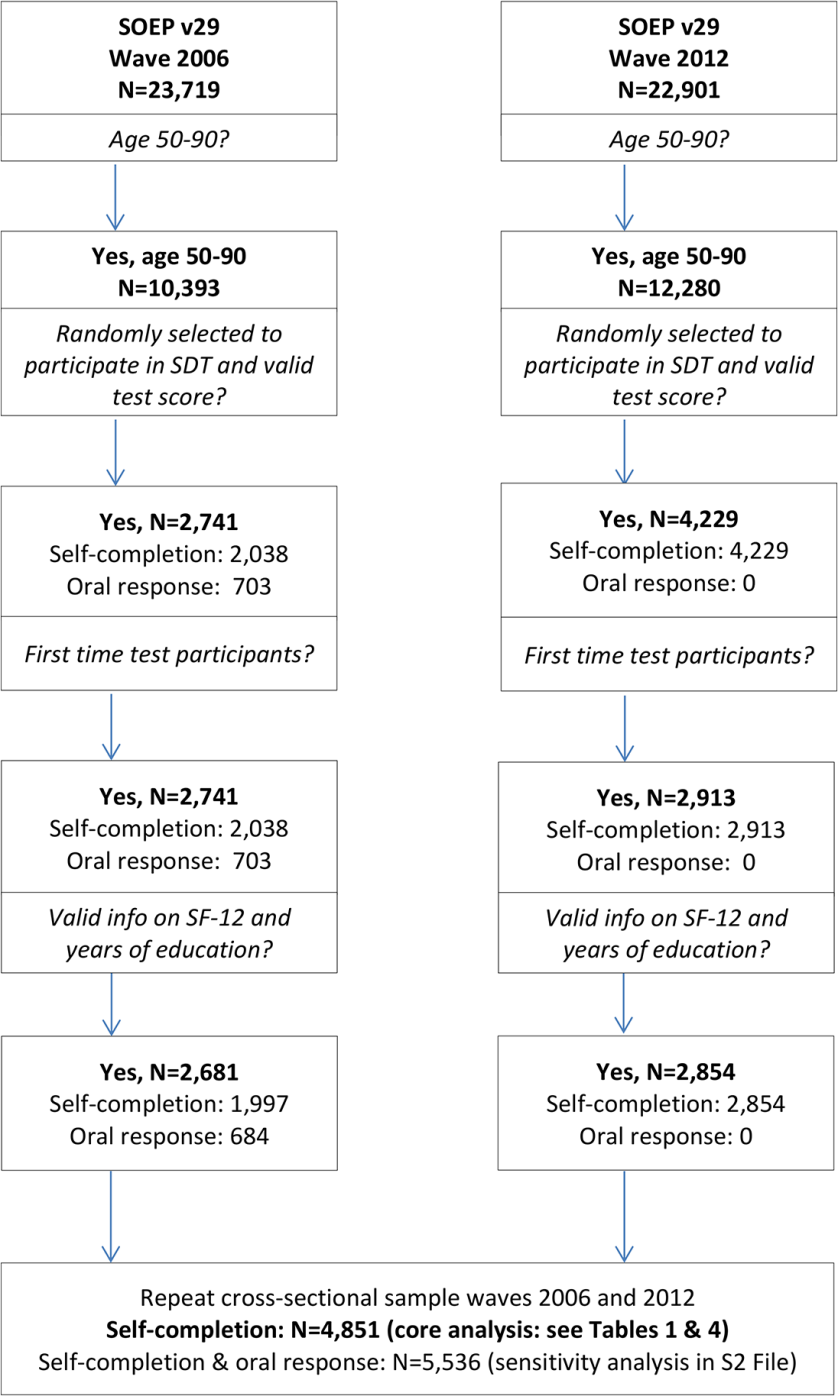
Sample Composition. Selection of participants for repeat cross-sectional analysis.

Sample composition: in 2006, a sample of 7,440 participants was randomly selected to participate in the cognitive tests. Almost 80% of those selected for participation provide valid test scores [33]. The 2006 net sample involves 5,545 participants. Many of these participants were again tested in 2012 and, in addition, a large refresher sample was tested for the first time in 2012. This study focuses on test participants aged 50–90 and, for methodological reasons, it only considers results from respondents’ first participation either in 2006 or 2012 (no repeat testing). As illustrated in a flowchart in Fig 1, the sample of analysis (after the application of the age restriction, random selection of cognitive test participants, and selection of first-time cognitive test participants) involves 2,741 individuals aged 50–90 in 2006 and 2,913 individuals aged 50–90 in 2012. About 98% of the sample members provide valid SF-12 health scores and full information on controls variables (sample size for both years, N = 5,536). Of these, 4,851 completed the SDT on a laptop (self-completion) and 684 gave oral responses (see Fig 1).

The analysis is carried out in three steps. First, to ascertain if individuals with better cognitive functioning tend to have a better physical and/or mental health status, *individual-level*, *cross-sectional correlations* between the SF-12 health measures and cognitive test scores (SDT) are calculated. These analyses are carried out separately for the two survey waves, based on samples of (1) individuals aged 50–90 in 2006 and (2) individuals aged 50–90 in 2012.

Second, based on a longitudinal sample of test participants in 2006 and 2012 (aged 50–90 in 2006), we investigate if changes in their cognitive functioning over time are associated with changes in their physical and/or mental health. To this end, we estimate change scores for individuals’ cognitive functioning, physical functioning, and mental health. The change score on one dimension of functioning/health is then regressed on the change scores on the other two dimensions while controlling for the level of functioning at baseline (in 2006).

Third, the core analysis in this study investigates population trends using a *repeat cross-sectional design*, i.e. using two independent samples of individuals aged 50–90, tested six years apart. To avoid upward bias in estimates of trends in average cognitive abilities due to re-testing effects [48], observations are restricted to the first test participation. The characteristics of our samples aged 50–90 for the two time points are summarized in Table 1. Using samples of individuals aged 50–90 from the two survey waves, we run OLS regressions using the continuous SDT, PCS, and MCS scores as outcome variables. The predictor of central interest is the time dummy indicating interview/testing in the earlier or later survey wave. It captures a blend of potential period and cohort effects. Most previous work with a similar design has interpreted it as a cohort effect [2,16,20]. In any case, the ‘time effect’ captures the degree to which respondents to the 2012 survey perform better or worse in the SDT–or report better or worse health–compared to respondents to the 2006 survey. The regressions estimate such time effects (trends), controlling for age and years of education. Moreover, to test if trends vary by age or education we estimate interactions between age, years of education, and time. Because of well-known gender-differences in population health and cognition [37,49], all analyses are carried out separately for women and men. To account for the possibility that the composite indicators PCS and MCS do not follow the same trend as the eight SF-12 subdomains [50–52], supplementary trend analyses are run for each of the subdomains (see S1 Table).

**Table 1:** Sample Characteristics.

	Men						Women					
	2006 (N = 969)			2012 (N = 1,376)			2006 (N = 1,028)			2012 (N = 1,478)		
	min	Max	mean (SD)	min	max	mean (SD)	min	max	mean (SD)	min	max	mean (SD)
**Age**	50	90	63.4	50	90	65.2	50	89	62.6	50	90	64.3
			(8.7)			(9.5)			(9.0)			(9.5)
**Years of education**	7	18	12.5	7	18	12.3	7	18	11.6	7	18	11.7
			(2.9)			(2.8)			(2.5)			(2.6)
**PCS**	13.9	68.8	47.8	13.0	68.4	46.0	14.6	70.1	45.8	13.1	67.9	44.9
			(9.8)			(10.0)			(10.8)			(10.6)
**MCS**	18.4	74.5	54.7	8.8	78.3	53.3	8.9	72.3	52.3	12.5	75.4	51.7
			(9.1)			(9.5)			(10.2)			(9.9)
**SDT**	21.5	71.8	45.0	21.5	75.8	46.9	21.5	69.8	44.5	21.5	70.8	46.3
			(9.0)			(8.2)			(9.1)			(8.1)

*Source*: German Socio-Economic Panel, pooled data for 2006 and 2012. *Abbreviations*: SDT—Symbol-Digit Test (cognition); PCS—composite score physical health; MCS—composite score mental health. The PCS, MCS, and SDT scores have been z-standardized with a mean of 50 and a SD of 10.

## Findings

### Individual-level associations

Cross-sectional correlation analyses confirm that those with better physical functioning tend to show better cognitive functioning (Table 2): For both men and women, we find a significant positive correlation between the SDT and the PCS scores. The strength of association is modest. The correlation coefficients for both sexes and survey years remain around 0.20. Mental health, by contrast, is largely unrelated to cognitive function. Moreover, the PCS and SDT scores show a positive correlation with hand-grip strength, suggesting that both measures are associated with individuals’ overall level of functioning.

**Table 2:** Cross-Sectional Correlations (Bivariate).

	Men		Women		N			
	2006	2012	2006	2012	M 2006	M 2012	W 2006	W 2012
**PCS and SDT**	0.18***	0.19***	0.17***	0.17***	969	1,376	1,028	1,478
**MCS and SDT**	0.05	0.02	0.03	0.05	969	1,376	1,028	1,478
**PCS and hand-grip**	0.33***	0.26***	0.37***	0.33***	1,081	1,485	1,153	1,539
**MCS and hand-grip**	0.04	0.06*	0.12**	0.07*	1,081	1,485	1,153	1,539
**SDT and hand-grip**	0.28***	0.25***	0.25***	0.25***	235	577	227	596

*Sample*: Women and men aged 50–90, cross-sectional samples for 2006 and 2012 waves. The samples for the correlations between the PCS, MCS, and SDT are those shown in Table 1 (respondents with valid data on all measures). The samples for the correlations with hand-grip strength are smaller because grip strength was only tested for a sub-sample of SOEP participants.

Significance levels

*** p<0.001

**p<0.01

*p<0.05.

Analyses that utilize the panel dimension of the data (longitudinal analyses) show that changes in older individuals’ cognitive functioning over time are associated with changes in their physical functioning (Table 3). When cognitive functioning and mental health scores improve, both changes are significantly associated with an improvement in physical functioning (Model 1). When physical functioning improves, so do mental health (Model 2) and cognitive functioning (Model 3). Changes in cognitive functioning are not associated with changes in mental health (Models 2 and 3).

**Table 3:** Longitudinal Associations: Change Score Analyses.

	Model 1 PF change score	Model 2 MH change score	Model 3 SDT change score
**PF change score**		0.16***	0.07**
**MH change score**	0.15***		0.00
**SDT change score**	0.11**	0.01	
**PF level 2006**	-0.51***	0.20***	0.13***
**MH level 2006**	0.18***	0.01	-0.03
**SDT level 2006**	0.19***	0.64***	-0.70***
**Constant**	7.83**	23.82	13.36
**N**	813	813	813
**Adj. R2**	0.24	0.33	0.42

*Sample*: Women and men aged 50–90, longitudinal sample for 2006 and 2012 waves. The sample excludes those giving oral responses in 2006. Change score = score in 2012 minus the score in 2006 for the same individual. Abbreviations: PF = physical functioning, MH = mental health, SDT = Symbol-Digit Test (cognition). The change score analysis draws on these SF-12 sub-dimensions instead of the composite indicators PCS and MCS, because the latter are orthogonal by construction, inducing a negative correlation of the change scores [48].

Significance levels

*** p<0.001

**p<0.01.

Overall, our findings at the *level of individuals* suggest a positive correlation of measures of cognitive and physical functioning both from a cross-sectional and a longitudinal perspective.

### Trends in population health

To investigate how the average level of functioning in the population aged 50–90 has changed over time (repeat cross-sectional design), we run a set of regressions of SDT, PCS, and MCS scores on age, education, and time. We find that the average cognitive functioning of those aged 50–90 and tested in 2012 is higher than that of the same age-group tested in 2006 (see Table 4, significant positive time effects on SDT for men and women). This indicates secular improvements in average cognitive abilities, referred to as *Flynn effects* in the literature [53]. At the same time, average PCS and MCS scores have declined (negative time effects), suggesting that population health in terms of cognitive functioning has not developed in parallel with population health in terms of physical functioning and mental well-being. The observed trends show differences by sex:

**Table 4:** Estimating Change in Average Functioning in the Population Aged 50–90.

***Men***	***SDT***	***PCS***	***MCS***
Age	-0.304***	-0.243***	1.037***
	(0.018)	(0.021)	(0.277)
Age squared			-0.007***
			(0.002)
Years of education	0.639***	0.706***	0.166*
	(0.058)	(0.070)	(0.068)
**Time**	**2.563** ***	**-1.241** **	**-1.463** ***
	(0.330)	(0.399)	(0.391)
Constant	56.289***	54.405***	15.665
	(1.391)	(1.684)	(9.069)
Observations	2,345	2,345	2,345
R-squared	0.169	0.103	0.023
***Women***	***SDT***	***PCS***	***MCS***
Age	-0.291***	-0.295***	0.797**
	(0.017)	(0.022)	(0.284)
Age squared			-0.005*
			(0.002)
Years of education	0.573***	0.768***	0.444***
	(0.063)	(0.081)	(0.080)
**Time**	**2.237** ***	**-0.483**	**-0.775**
	(0.320)	(0.410)	(0.407)
Constant	56.097***	55.416***	19.040*
	(1.444)	(1.849)	(9.246)
Observations	2,506	2,506	2,506
R-squared	0.163	0.121	0.018

*Sample*: First-time participants in cognitive testing in the SOEP in 2006 or 2012. Population aged 50–90 at the time of interview. *Abbreviations*: SDT—Symbol-Digit Test (cognition); PCS—composite score physical health; MCS—composite score mental health. The PCS, MCS, and SDT scores have been z-standardized with a mean of 50 and a SD of 10.

Significance levels

*** p<0.001

**p<0.01

*p<0.05.

Regressions using the eight SF-12 sub-dimensions as outcome variables are shown in S1 Table.

Among men, cognitive functioning (SDT) has on average improved by 2.6 score points (which correspond to 0.26 SD) within the 6-year period. At the same time, we find negative health trends in terms of physical and mental health (PCS: -0.12 SD; MCS: -0.15 SD). For women, we find a significant Flynn effect of a similar magnitude as for men (SDT: +0.22 SD, in a pooled model for men and women, the interaction between sex and time is found to be non-significant), whereas average mental and physical health show negative trends that fall short of statistical significance. Supplementary analyses with the SF-12 sub-domains show a significant negative trend on the sub-indicator measuring role limitations due to physical health problems (RP:- 0.14 SD) also for women (see S1 Table). In sum, we find similar positive trends in terms of cognition for men and women but sex-differentiated trends in terms of physical and mental health. For men, the physical and mental health decline is found to be stronger.

**Fig 2:**
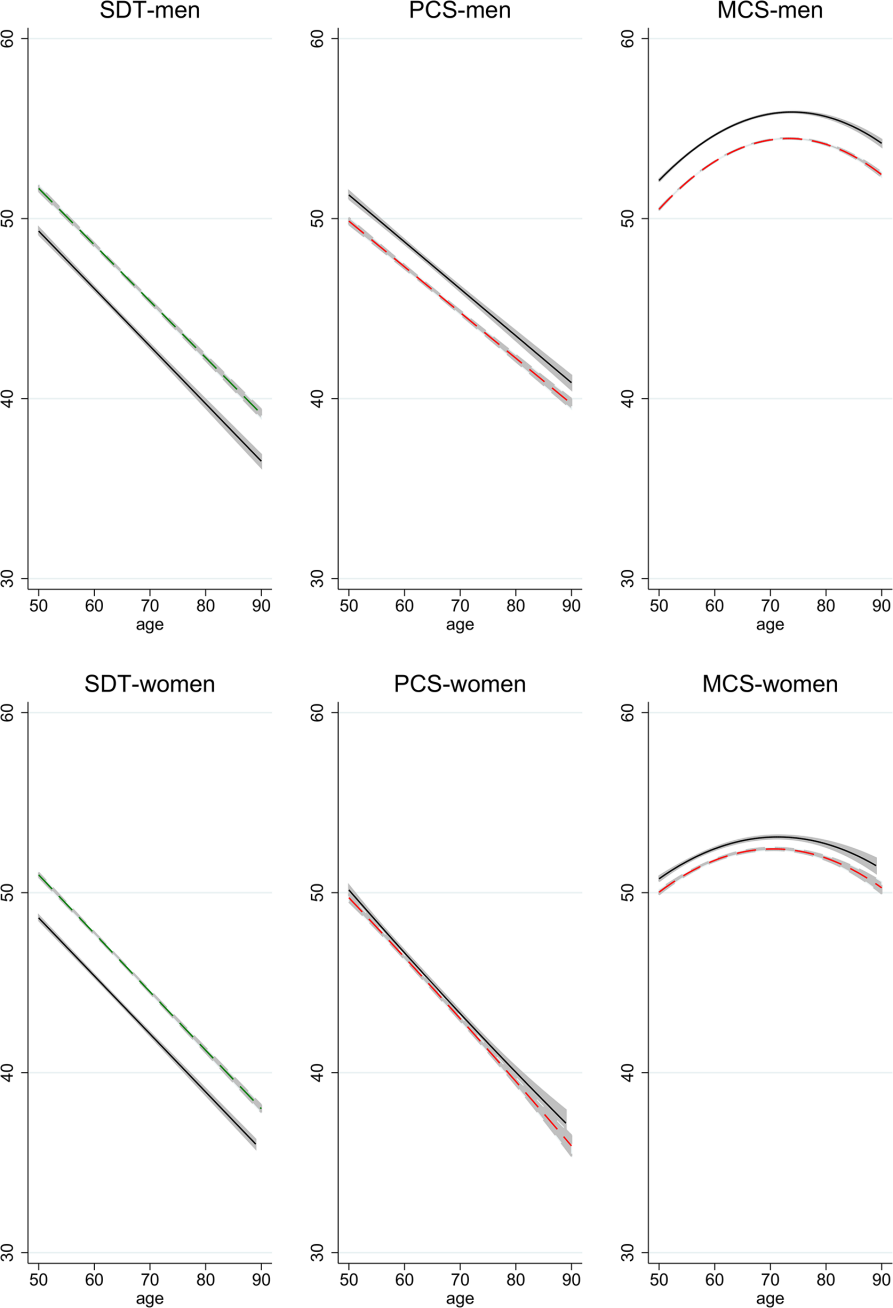
Age-Profiles of Functioning in 2006 and 2012. Age-profiles in 2006 (solid) and 2012 (dashed), predicted based on regressions that control for age, age squared where significant at p<0.05, education, and time. Confidence intervals for predicted means in each year.

These generalized findings of *population health trends* are illustrated in Fig 2, which shows predicted age-profiles of functioning for 2006 (solid line) and 2012 (dashed line). The regressions underlying the predicted profiles include age, age squared where significant, years of education, and time as predictors (cf. Table 4). Fig 2 shows that SDT and PCS scores decline with age in a fairly linear fashion, while MCS scores show an inverse U-shaped relation with age. The distances between the two age-profiles reflect the time effects, showing a positive trend in terms of the SDT but a negative one in terms of PCS and MCS (especially for men).

### Population sub-groups

To investigate if population trends vary across population sub-groups, we estimate results separately for three age-groups (50–64, 65–74, and 75–90). Moreover, we distinguish three levels of education. Low education is defined as having completed a general elementary or basic vocational qualification at most. An intermediate level of education denotes completion of an intermediate general or vocational qualification including those who have obtained a general or vocational maturity certificate. A high level of education pertains to the completion of a tertiary degree.

Our findings for the different sub-groups of the population are summarized in Table 5, which shows only the coefficients pertaining to the time effects of interest (the coefficients for the control variables are omitted). The positive trend in terms of cognition (SDT) is found to be significant for all population groups: women and men in all age groups and at all levels of education (interaction effects of age and education with time are not significant). By contrast, physical health and mental health show group-specific trends:

**Table 5:** Time Effects for Population Subgroups.

***Men***	***SDT***	***PCS***	***MCS***	**N**
Age 50–90	2.563***	-1.241**	-1.463***	2,345
Age 50–64	2.737***	-2.098***	-1.736**	1,206
Age 65–74	2.133***	-0.736	-1.076	779
Age 75–90	2.761***	1.303	-0.744	360
Low education	2.819***	-1.865**	-1.187*	1,159
Intermediate edu	1.950**	-1.458	-1.779*	551
High education	2.656***	-0.117	-1.801**	662
Age 50–64, low edu	2.892***	-3.719***	-1.866*	528
Age 50–64, med/hi edu	2.610***	-1.005	-1.747*	689
*Women*	***SDT***	***PCS***	***MCS***	N
Age 50–90	2.237***	-0.483	-0.775	2,506
Age 50–64	1.859***	-1.510**	-1.393*	1,389
Age 65–74	2.559***	1.475	0.774	761
Age 75–90	3.324***	0.126	-1.453	356
Low education	2.244***	0.284	-0.758	1,298
Intermediate edu	1.596**	-1.092	-0.926	811
High education	2.839***	-2.366**	-0.771	418
Age 50–64, low edu	1.392*	-1.223	-1.871*	581
Age 50–64, med/hi edu	2.041***	-1.856**	-1.130	818

*Sample*: First-time participants in cognitive testing in the SOEP in 2006 or 2012. Population aged 50–90 at the time of interview. Regression analyses run for separate population groups and outcome measures; coefficients show time effects (2012 vs. 2006), controlling for age, age squared, and years of education. *Abbreviations*: SDT—Symbol-Digit Test (cognition); PCS—composite score physical health; MCS—composite score mental health. The PCS, MCS, and SDT scores have been z-standardized with a mean of 50 and a SD of 10.

Significance levels

*** p<0.001

**p<0.01

*p<0.05.

Regressions using the eight SF-12 sub-dimensions as outcome variables are shown in S1 Table.

For men and women, PCS and MCS scores have declined mainly for those aged 50–64, whereas less evidence for health declines is found for the older parts of the population. For men aged 75+ and for women aged 65+, trend in average PCS scores even point to a health improvement (if not statistically significant). The results furthermore suggest that whereas trends in cognitive function are fairly similar across educational levels, trends in physical and mental health vary across education levels in a rather complex fashion (Table 5). Some of the health declines are restricted to men at low levels of education (in terms of PCS). MCS scores have decreased for men at all levels of education. In the case of women, negative trends in terms of physical health are only found for the more highly educated.

The population-group for which the most strongly diverging trends are found are men aged 50–64 and at low levels of education (Table 5). For this group, the physical health decline over the 6-year interval is estimated to amount to about 0.37 SD in terms of PCS and 0.19 SD in terms of MCS, while cognitive functioning (SDT) increased by 0.29 SD.

### Fully interacted models

**Fig 3:**
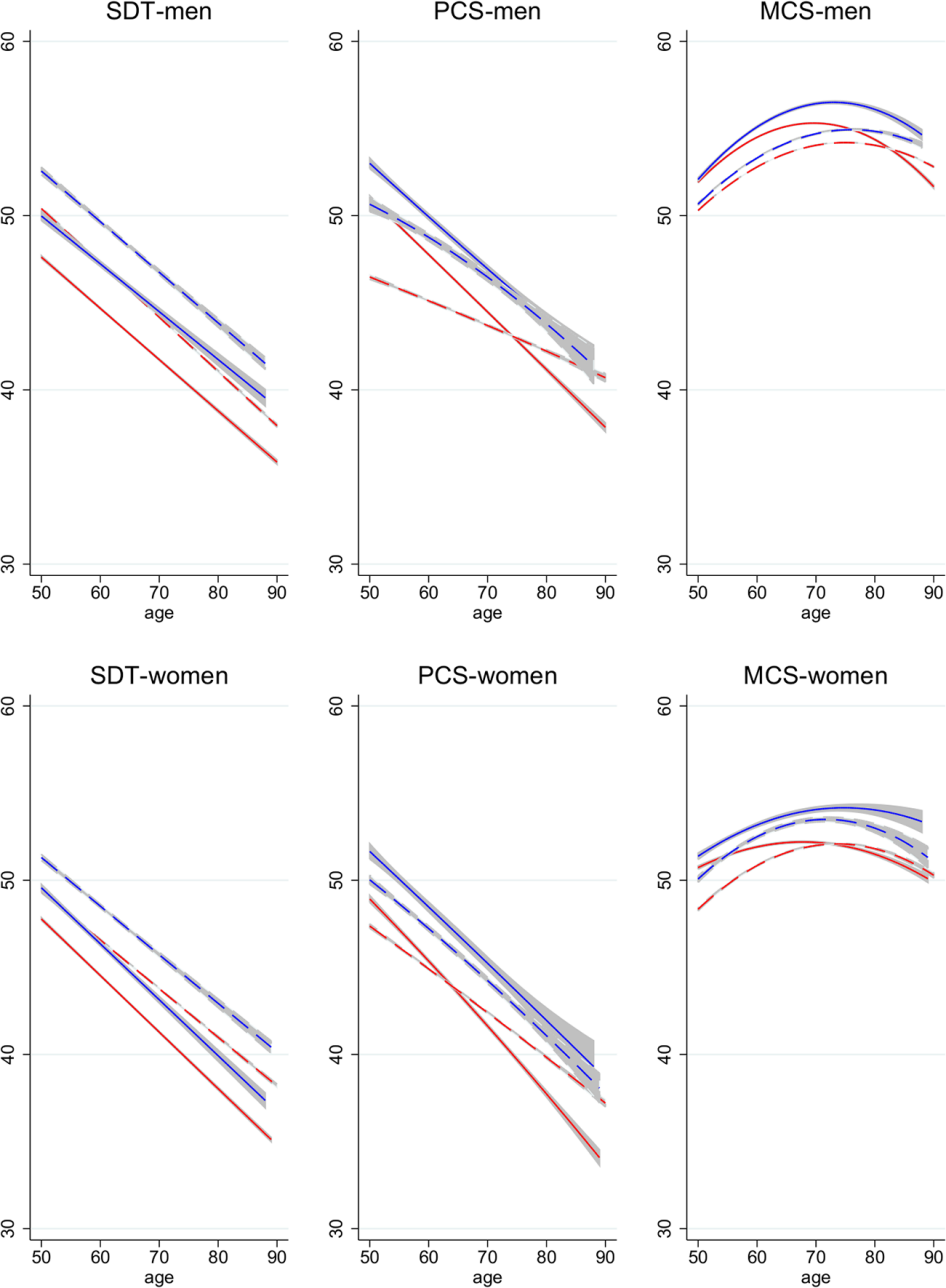
Age-Profiles of Functioning in 2006 and 2012, by Education. Age-profiles in 2006 (solid) and 2012 (dashed) for low-educated (red) and medium/high-educated (blue) population aged 50–90. Profiles predicted based on regressions that involve full interactions of age, age-squared, education, and time. Confidence intervals for predicted means in each year.

For a more formal test of different trends across population sub-groups, we test for the significance of interaction effects of the time dummy with education and age (and age squared) and for the interaction of age with education. The results from these analyses are graphically presented in Fig 3 that shows predicted *age-profiles of functioning* separately for the two time points (solid for 2006, dashed for 2012) and two levels of education (red for low education and blue for medium/high education). The results illustrated in Fig 3 resemble those shown in Table 5 but are not identical due to the inclusion of full interactions of age, education, and time.

Fig 3 shows that cognitive functioning and physical health tend to be better at higher levels of education (the blue lines are generally higher than the red lines pertaining to the same year). Moreover, they show that cognitive and physical functioning tends to decline with age–both for the low-educated and the medium/high-educated. In case of the SDT, the age-profiles for 2006 and 2012 are roughly parallel, illustrating a Flynn effect of similar magnitude across ages (and a Flynn effect at both education levels). This finding holds for men and women.

In contrast to the parallel age-cognition-profiles for 2006 and 2012, the age-profiles for low-educated men’s PCS scores cross at around age 75, illustrating the different trends for different age groups: whereas average health declined for the young-old, it improved for the old-old. The general pattern of larger physical health declines for the young-old than the old-old holds for both education groups. However, the health decline for young-old men is stronger for the low-educated (red profiles). For medium/high-educated men, small health declines are found at younger ages and a stable trend at higher ages (no crossing of blue lines). The age-profiles differentiated by educational attainment for the mental health measures are more difficult to interpret given their U-shaped relation with age. However, the general pattern of larger declines in average health at younger ages is also shown for the mental health measures. The age-profiles of functioning for women are similar to those of men insofar as the profiles for the lower educated are generally at a lower level. Moreover, the physical health profiles for low-educated women cross, indicating a more positive trend at higher ages.

The general conclusions remain the same irrespective of whether or not those who had given oral responses in 2006 are included: cognitive functioning shows a secular improvement for all age groups and at all educational levels (significant Flynn effects), whereas physical and mental health declined to a significant degree among young-old men and women (age 50–64) and in particular for low educated, young-old men (see S2 File for robustness analyses).

## Discussion

Research on the health of aging populations has shown that age-related declines in physical functioning (e.g., mobility limitations, declining strength and speed of movement) are associated with declines in cognitive functioning. A large number of studies, including the present one, show that those with better physical functioning tend to score higher in tests of cognitive functioning (positive individual-level cross-sectional correlations). Moreover, from a longitudinal perspective, prior studies, as well as the present one, report that individuals whose physical functioning declines also tend to show cognitive decline, and *vice versa* (positive individual-level longitudinal associations), suggesting that cognitive and physical functioning interact within an *age-related cycle of decline* [54]. Based on findings of this kind, a prominent hypothesis in aging research is the existence of a common cause that co-determines aging processes in cognitive and physical terms. One prediction that may but need not necessarily follow from the *common cause assumption* is that trends in the average physical health of populations tend to go in the same direction as trends in the average cognitive functioning of populations. While the scant available research to date would support such contentions for the aging population in Denmark [2] and Finland [9], the present study finds *diverging trends* in the average level of functioning of the German population aged 50–90 across different domains. The study finds a positive trend in the average cognitive functioning of the population and a negative trend in the average physical functioning of the same population.

How can trends in average cognitive and physical functioning go in different directions? A plausible explanation is based on the view that functional decline results from a lack of training (use-it-or-lose-it). Physical inactivity is an important cause of most chronic diseases [55], while a lack of cognitive activity increases the risk for cognitive decline [56,57]. From this perspective, our results may be explained by *changing life styles*. Life may have become cognitively more demanding while involving declining levels of physical activity. Daily lives may involve *increasing levels of complexity* given the spread of modern technology across all walks of life that challenges users with frequent product innovations and changing user interfaces (e.g., smart devices with touch screens and internet connection). Moreover, the cognitive *speed of life* may have increased with the spread of modern technology. Indeed, supplementary analyses of the SOEP data (S1 Fig) show that in the older German population, and in particular at ages 65 and over, the use of computers and mobile phones has significantly increased during the observation period (see also [13]). Furthermore, more recent cohorts of the German population are more likely to work until higher ages (S1 Fig), which may also contribute to the maintenance of cognitive function. At the same time, for many people daily routines involve declining levels of physical activity as evidenced by rising levels of obesity in Germany [58]. Many jobs have become more complex and cognitively challenging [59] while involving declining levels of health-beneficial physical activity (e.g., sedentary computer work). Prior research confirms that occupational complexity in mid-life helps maintain good cognitive functioning in old age [57,60–62] while ‘occupational sitting’ increases physical health risks [63].

Another notable finding of this study is that the observed secular improvement in cognitive functioning is of similar magnitude in all age groups. As recently pointed out by Salthouse [19], this may be taken as evidence that the Flynn effect represents a period effect rather than a cohort effect. Period effects affect all individuals regardless of their age, while cohort effects only affect certain age groups but persist over time.

In any case, the finding of similar Flynn effects for all age groups stands in interesting contrast to the age-graded trends in physical health found in this study. The analyses suggest that average physical health declined in the young-old German population (age 50–64) and to a lesser extent for those aged 65–74, while the physical performance of the oldest-old (age 75–90) may even have improved on some dimensions. Extrapolating to higher ages, this is in line with previous findings of favorable cohort trends among nonagenarians [2, 54]. Rising levels of disability in more recent cohorts of young-olds have before been shown for France [64] and England [65]. Moreover, recent studies based on US American data show a similar picture concerning age-graded trends: Seeman et al. [66] report an increasing prevalence of reported disability between the late 1980s and the early 2000s for the young-old (aged 60–69) and a stable trend for those aged 70–79 years, whereas for those aged 80 years and older they present evidence of declines in functional limitations. Also Martin and Schoeni [67] show evidence of increases in functional limitations for those aged 40–64, whereas for those aged 65 years and older the prevalence of limitations has declined.

Moreover, we find different trends for different education groups–not in terms of cognitive functioning but in terms of physical functioning. The comparatively strongest decline is observed for young-old men at low levels of education. Most other studies in the field have not investigated trend heterogeneity by socio-economic status, except for research based on US data that also finds increases in ADL disability prevalence among the elderly for the lowest income and education groups but not for more advantaged groups [68] and a study based on English data for the population aged 65 and older, showing worse trends for mobility functions in the lower educated population [69]. A potential explanation for education-graded health trends in the framework of the ‘changing lifestyle’ argument is that higher educated individuals are more likely to adopt health behaviors (e.g., regular physical exercise) that compensate for sedentary occupations (i.e. educated-graded period effect). Other explanations may lie in the different occupational histories and life styles of lower and higher educated groups prior to the spread of modern technology and computerized work (i.e. cohort effect).

A potential limitation of studies of the kind carried out here is that survey participation is more difficult for demented or severely disabled individuals who are thus excluded from the analysis. Estimated trends in average functioning would be biased if the composition of the two cross-sectional samples changed over time concerning the share of demented individuals, for example. Doblhammer et al. [70] show that during the observation period the prevalence of dementia has slightly declined among German women aged 75–84, while no significant trend in dementia prevalence was observed for men of this age. In consequence, we may slightly overestimate women’s average cognitive functioning in 2006 and, in turn, underestimate the positive trend in cognition. As pointed out previously by other scholars, the representativeness of changes in sample composition represents a potential limitation to all survey-based studies of population health trends (e.g., [65] pointing out that people in institutional care tend to be excluded from most surveys and that the share of such individuals may change over time). In any case, potential concerns of this kind do not limit the central conclusion from this study: using the exact same two cross-sectional population samples (individuals aged 50–90 in 2006 and individuals aged 50–90 in 2012) for trend analyses of cognitive, physical and mental health, *diverging trends* in the different health dimensions are observed.

The findings from the present study corroborate the view that aging, i.e. age-associated functional decline, is a *multi-dimensional* phenomenon. Higher chronological age is a strong predictor of declines in cognitive and physical functioning, old-age probably being the single most important risk factor for dementia and frailty [54]. However, functional decline is age-associated not age-determined and there is evidence that aging processes can be delayed if older people keep on doing physical exercise and continue challenging their brains. If they stop doing one or the other, functioning in one domain (e.g., physical fitness) may decline faster than in others (cognition). Age-associated functional decline can be stronger in some domains than others. In some people, cognitive decline may in fact be entirely decoupled from physical functioning. In a recent study of Alzheimer’s dementia patients, 22% showed no signs of physical frailty [71]. Vice versa, a minority of physically frail people show signs of cognitive impairment [54]. As a result, individual-level correlations between measures of cognitive and physical functioning tend to be positive, yet rather weak. In a similar vein, conclusions about population health trends are contingent on the health measures used. While an aging population may show falling levels of average physical health, the same population may exhibit increasing levels of cognitive functioning and thus ‘age’ more successfully in the cognitive than the physical domain. A common factor driving such diverging trends may be modern technology that increases cognitive challenges in everyday life and work while reducing incentives and the necessity for physical activity. In the longer run, technology that fosters a sedentary behavior may threaten cognitive health since physical activity is important for the maintenance of cognitive functioning [72]. However, modern technology may also play a protective role in this regard, if it allows for the cognitive stimulation of those who are physically constrained from participating in stimulating activities (e.g., work, social interaction). Moreover, technology may help to render physical limitations less disabling and hence reduce mobility limitations in older populations, allowing them to stay engaged and cognitively active.

## Supporting Information

S1 Fig Societal Change: Modern Technology and Paid Work Integration. Source: SOEP. Values refer to the share of respondents aged 50–90 who have a personal computer or who have a mobile phone in the household. Increases in the use of PCs/mobiles are significant for women and men and for both age groups (p<0.001). Increases in rates of paid work participation are significant for women in both age groups and men aged 50–64 (p<0.01).
(TIF)

S1 File Description of SF-12 Measures Included in the SOEP. (DOCX)

S2 File Sensitivity Analyses Including Those Giving Oral Responses in 2006. (DOCX)

S1 Table Time Effects for Population Subgroups, SF-12 Sub-Dimensions. *Sample*: First-time participants in cognitive testing in the SOEP in 2006 or 2012. Population aged 50–90 at the time of interview. Regression analyses run for separate population groups and outcome measures; coefficients show time effects (2012 vs. 2006), controlling for age, age squared, and years of education. *Abbreviations*: PF–physical functioning; RP–role physical; BP–bodily pain; GH–general health; VT–vitality; SF–social functioning; RE–role emotional; MH–mental health (for details on survey questionnaire wording, see S1 File).
(DOCX)

## References

[1] Christensen K, Doblhammer G, Rau R, Vaupel JW. Ageing populations: the challenges ahead. Lancet. 2009;374: 1196–1208. 10.1016/S0140-6736(09)61460-4 19801098 PMC2810516

[2] Christensen K, Thinggaard M, Oksuzyan A, Steenstrup T, Andersen-Ranberg K, Jeune B, et al Physical and cognitive functioning of people older than 90 years: a comparison of two Danish cohorts born 10 years apart. Lancet. 2013;382: 1507–1513. 10.1016/S0140-6736(13)60777-1 23849796 PMC3818336

[3] Crimmins EM. Trends in the health of the elderly. Annu Rev Public Health. 2004;25: 79–98. 10.1146/annurev.publhealth.25.102802.124401 15015913

[4] Fries JF. Aging, natural death, and the compression of morbidity. N Engl J Med. 1980;303: 130–135. 10.1056/NEJM198007173030304 7383070

[5] Kluge F, Zagheni E, Loichinger E, Vogt T. The advantages of demographic change after the wave: fewer and older, but healthier, greener, and more productive? PLoS ONE. 2014;9: e108501 10.1371/journal.pone.0108501 25250779 PMC4177216

[6] Rowe JW, Kahn RL. Human aging: usual and successful. Science. 1987;237: 143–149. 3299702 10.1126/science.3299702

[7] Vaupel JW. Biodemography of human ageing. Nature. 2010;464: 536–542. 10.1038/nature08984 20336136 PMC4010874

[8] Falk H, Johansson L, Ostling S, Thøgersen Agerholm K, Staun M, Høst Dørfinger L, et al Functional disability and ability 75-year-olds: a comparison of two Swedish cohorts born 30 years apart. Age Ageing. 2014;43: 636–641. 10.1093/ageing/afu018 24595067

[9] Heikkinen E, Kauppinen M, Rantanen T, Leinonen R, Lyyra T-M, Suutama T, et al Cohort differences in health, functioning and physical activity in the young-old Finnish population. Aging Clin Exp Res. 2011;23: 126–134. 10.3275/6932 20308805

[10] Manton KG. Recent declines in chronic disability in the elderly U.S. population: risk factors and future dynamics. Annu Rev Public Health. 2008;29: 91–113. 10.1146/annurev.publhealth.29.020907.090812 18031222

[11] Pérès K, Edjolo A, Dartigues J-F, Barberger-Gateau P. Recent trends in disability-free life expectancy in the French elderly twenty years follow-up of the paquid cohort. Annu Rev Gerontol Geriatr. 2013;33: 293–311. 10.1891/0198-8794.33.293

[12] Baxendale S. The Flynn effect and memory function. J Clin Exp Neuropsychol. 2010;32: 699–703. 10.1080/13803390903493515 20119877

[13] Bordone V, Scherbov S, Steiber N. Smarter every day: the deceleration of population ageing in terms of cognition. Intelligence. 2015; Article in Press. 10.1016/j.intell.2015.07.005

[14] Finkel D, Reynolds CA, McArdle JJ, Pedersen NL. Cohort differences in trajectories of cognitive aging. J Gerontol B Psychol Sci Soc Sci. 2007;62: P286–294. 17906170 10.1093/geronb/62.5.p286

[15] Gerstorf D, Ram N, Hoppmann C, Willis SL, Schaie KW. Cohort differences in cognitive aging and terminal decline in the Seattle Longitudinal Study. Dev Psychol. 2011;47: 1026–1041. 10.1037/a0023426 21517155 PMC3134559

[16] Rönnlund M, Nilsson L-G. The magnitude, generality, and determinants of Flynn effects on forms of declarative memory and visuospatial ability: Time-sequential analyses of data from a Swedish cohort study. Intelligence. 2008;36: 192–209. 10.1016/j.intell.2007.05.002

[17] de Rotrou J, Wu Y-H, Mabire J-B, Moulin F, de Jong LW, Rigaud A-S, et al Does cognitive function increase over time in the healthy elderly? PLoS ONE. 2013;8: e78646 10.1371/journal.pone.0078646 24244332 PMC3823862

[18] Sacuiu S, Gustafson D, Sjögren M, Guo X, Östling S, Johansson B, et al Secular changes in cognitive predictors of dementia and mortality in 70-year-olds. Neurology. 2010;75: 779–785. 10.1212/WNL.0b013e3181f0737c 20805523 PMC2938967

[19] Salthouse TA. Implications of the Flynn effect for age-cognition relations. Intelligence. 2015;48: 51–57. 10.1016/j.intell.2014.10.007 25506107 PMC4260335

[20] Skirbekk V, Stonawski M, Bonsang E, Staudinger UM. The Flynn effect and population aging. Intelligence. 2013;41: 169–177. 10.1016/j.intell.2013.02.001

[21] Llewellyn DJ, Matthews FE. Increasing levels of semantic verbal fluency in elderly English adults. Aging Neuropsychol Cogn. 2009;16: 433–445. 10.1080/13825580902773867 19319746

[22] Matthews FE, Arthur A, Barnes LE, Bond J, Jagger C, Robinson L, et al A two-decade comparison of prevalence of dementia in individuals aged 65 years and older from three geographical areas of England: results of the Cognitive Function and Ageing Study I and II. The Lancet. 2013;382: 1405–1412. 10.1016/S0140-6736(13)61570-6 PMC3906607 23871492

[23] Sanderson WC, Scherbov S. The characteristics approach to the measurement of population aging. Popul Dev Rev. 2013;39: 673–685. 10.1111/j.1728-4457.2013.00633.x

[24] Crimmins EM, Beltrán-Sánchez H. Mortality and morbidity trends: is there compression of morbidity? J Gerontol B Psychol Sci Soc Sci. 2011;66B: 75–86. 10.1093/geronb/gbq088 PMC3001754 21135070

[25] Lafortune G, Balestat G. Trends in severe disability among elderly people: assessing the evidence in 12 OECD vountries and the future implications. OECD Health Work Pap. 2007; No. 26. 10.1787/217072070078

[26] Cambois E, Clavel A, Romieu I, Robine J-M. Trends in disability-free life expectancy at age 65 in France: consistent and diverging patterns according to the underlying disability measure. Eur J Ageing. 2008;5: 287–298. 10.1007/s10433-008-0097-1 28798581 PMC5546295

[27] Christensen H, Mackinnon AJ, Korten A, Jorm AF. The “common cause hypothesis” of cognitive aging: Evidence for not only a common factor but also specific associations of age with vision and grip strength in a cross-sectional analysis. Psychol Aging. 2001;16: 588–599. 10.1037/0882-7974.16.4.588 11766914

[28] Clouston SAP, Brewster P, Kuh D, Richards M, Cooper R, Hardy R, et al The dynamic relationship between physical function and cognition in longitudinal aging cohorts. Epidemiol Rev. 2013;[Epub ahead of print]. 10.1093/epirev/mxs004 PMC3578448 23349427

[29] Kuh D, Karunananthan S, Bergman H, Cooper R. A life-course approach to healthy ageing: maintaining physical capability. Proc Nutr Soc. 2014;73: 237–248. 10.1017/S0029665113003923 24456831 PMC3981474

[30] Robinson WS. Ecological correlations and the behavior of individuals. Am Sociol Rev. 1950;15: 351–357.

[31] Eibich P, Ziebarth NR. Examining the structure of spatial health effects in Germany using Hierarchical Bayes Models. Reg Sci Urban Econ. 2014;49: 305–320. 10.1016/j.regsciurbeco.2014.06.005

[32] Hays RD, Sherbourne CD, Mazel R. User’s manual for the medical outcomes study (MOS) core measures of health-related quality of life. RAND Monogr Rep. 1995;MR-162-RC. Available: http://www.rand.org/pubs/monograph_reports/MR162.html

[33] Ware J, Kosinski M, Keller SD. A 12-item short-form health survey: construction of scales and preliminary tests of reliability and validity. Med Care. 1996;34: 220–233. 8628042 10.1097/00005650-199603000-00003

[34] Andersen HH, Mühlbacher A, Nübling M, Schupp J, Wagner GG. Computation of standard values for physical and mental health scale scores using the SOEP version of SF12v2. Schmollers Jahrb. 2007;127: 171–182.

[35] Gill SC, Butterworth P, Rodgers B, Mackinnon A. Validity of the mental health component scale of the 12-item Short-Form Health Survey (MCS-12) as measure of common mental disorders in the general population. Psychiatry Res. 2007;152: 63–71. 10.1016/j.psychres.2006.11.005 17395272

[36] Vilagut G, Forero CG, Pinto-Meza A, Haro JM, de Graaf R, Bruffaerts R, et al The mental component of the Short-Form 12 Health Survey (SF-12) as a measure of depressive disorders in the general population: results with three alternative scoring methods. Value Health. 2013;16: 564–573. 10.1016/j.jval.2013.01.006 23796290

[37] Fleishman JA, Lawrence WF. Demographic variation in SF-12 scores: true differences or differential item functioning? Med Care. 2003;41: III75–III86. 12865729 10.1097/01.MLR.0000076052.42628.CF

[38] Franks P, Gold MR, Fiscella K. Sociodemographics, self-rated health, and mortality in the US. Soc Sci Med 1982. 2003;56: 2505–2514. 12742613 10.1016/s0277-9536(02)00281-2

[39] Lacson E, Xu J, Lin S-F, Dean SG, Lazarus JM, Hakim RM. A comparison of SF-36 and SF-12 composite scores and subsequent hospitalization and mortality risks in long-term dialysis patients. Clin J Am Soc Nephrol CJASN. 2010;5: 252–260. 10.2215/CJN.07231009 20019120 PMC2827595

[40] Burdine JN, Felix MR, Abel AL, Wiltraut CJ, Musselman YJ. The SF-12 as a population health measure: an exploratory examination of potential for application. Health Serv Res. 2000;35: 885–904. 11055454 PMC1089158

[41] Ambrasat J, Schupp J, Wagner GG. Comparing the predictive power of subjective and objective health indicators: changes in hand grip strength and overall satisfaction with life as predictors of mortality [Internet]. 2011. Report No.: SOEPpapers No. 398. Available: http://www.diw.de/documents/publikationen/73/diw_01.c.378114.de/diw_sp0398.pdf

[42] Bohannon RW. Hand-grip dynamometry predicts future outcomes in aging adults. J Geriatr Phys Ther 2001. 2008;31: 3–10. 18489802 10.1519/00139143-200831010-00002

[43] Cooper RS, Bjørn Heine Hardy, Rebecca Patel, Kushang V Kuh, Diana. Physical capability in mid-life and survival over 13 years of follow-up: British birth cohort study. BMJ. 2014;348 10.1136/bmj.g2219 PMC4004787 24787359

[44] Smith A. Symbol digits modalities test Los Angeles, CA: Western Psychological Services; 1982.

[45] Heineck G, Anger S. The returns to cognitive abilities and personality traits in Germany. Labour Econ. 2010;17: 535–546.

[46] Lang F, Weiss D, Stocker A, Rosenbladt B von. Assessing cognitive capacities in computer-assisted survey research: two ultra-short tests of intellectual ability in the German Socio-Economic Panel (SOEP). Schmollers Jahrb. 2007;127: 183–192.

[47] Schupp J, Herrmann S, Jaensch P, Lang FR. Erfassung kognitiver Leistungspotentiale Erwachsener im Sozio-oekonomischen Panel (SOEP) [Internet]. DIW Berlin; 2008. Available: http://www.diw.de/documents/publikationen/73/diw_01.c.85173.de/diw_datadoc_2008-032.pdf

[48] Ferrer E, Salthouse TA, Stewart WF, Schwartz BS. Modeling age and retest processes in longitudinal studies of cognitive abilities. Psychol Aging. 2004;19: 243–259. 10.1037/0882-7974.19.2.243 15222818

[49] Weber D, Skirbekk V, Freund I, Herlitz A. The changing face of cognitive gender differences in Europe. Proc Natl Acad Sci. 2014;111: 11673–11678. 10.1073/pnas.1319538111 25071201 PMC4136621

[50] Fleishman JA, Selim AJ, Kazis LE. Deriving SF-12v2 physical and mental health summary scores: a comparison of different scoring algorithms. Qual Life Res. 2010;19: 231–241. 10.1007/s11136-009-9582-z 20094805

[51] Mishra GD, Hockey R, Dobson AJ. A comparison of SF-36 summary measures of physical and mental health for women across the life course. Qual Life Res. 2014;23: 1515–1521. 10.1007/s11136-013-0586-3 24297102

[52] Parker MG, Thorslund M. Health trends in the elderly population: getting better and getting worse. The Gerontologist. 2007;47: 150–158. 17440120 10.1093/geront/47.2.150

[53] Hiscock M. The Flynn effect and its relevance to neuropsychology. J Clin Exp Neuropsychol. 2007;29: 514–529. 10.1080/13803390600813841 17564917

[54] Robertson DA, Savva GM, Kenny RA. Frailty and cognitive impairment—A review of the evidence and causal mechanisms. Ageing Res Rev. 2013;12: 840–851. 10.1016/j.arr.2013.06.004 23831959

[55] Booth FW, Roberts CK, Laye MJ. Lack of exercise is a major cause of chronic diseases. Compr Physiol. 2012;2: 1143–1211. 10.1002/cphy.c110025 23798298 PMC4241367

[56] Hultsch DF, Hertzog C, Small BJ, Dixon RA. Use it or lose it: engaged lifestyle as a buffer of cognitive decline in aging? Psychol Aging. 1999;14: 245–263. 10403712 10.1037//0882-7974.14.2.245

[57] Marioni RE, Valenzuela MJ, van den Hout A, Brayne C, Matthews FE. Active cognitive lifestyle is associated with positive cognitive health transitions and compression of morbidity from age sixty-five. PLoS ONE. 2012;7: e50940 10.1371/journal.pone.0050940 23251404 PMC3521012

[58] Ruesten A von, Steffen A, Floegel A, van der A DL, Masala G, Tjønneland A, et al Trend in obesity prevalence in European adult cohort populations during follow-up since 1996 and their predictions to 2015. PLoS ONE. 2011;6: e27455 10.1371/journal.pone.0027455 22102897 PMC3213129

[59] Oesch D. Occupational change in Europe: how technology and education transform the job structure Oxford University Press; 2013.

[60] Andel R, Silverstein M, Kåreholt I. The role of midlife occupational complexity and leisure activity in late-life cognition. J Gerontol B Psychol Sci Soc Sci. 2014;[Epub ahead of print]. 10.1093/geronb/gbu110 25190210

[61] Bosma H, van Boxtel MPJ, Ponds RWHM, Houx PJ, Burdorf A, Jolles J. Mental work demands protect against cognitive impairment: MAAS prospective cohort study. Exp Aging Res. 2003;29: 33–45. 10.1080/03610730303710 12735080

[62] Ribeiro PCC, Lopes CS, Lourenço RA. Complexity of lifetime occupation and cognitive performance in old age. Occup Med. 2013;63: 556–562. 10.1093/occmed/kqt115 24253807

[63] Marshall S, Gyi D. Evidence of health risks from occupational sitting. Am J Prev Med. 2010;39: 389–391. 10.1016/j.amepre.2010.07.001 20837292

[64] Cambois E, Blachier A, Robine J-M. Aging and health in France: an unexpected expansion of disability in mid-adulthood over recent years. Eur J Public Health. 2013;23: 575–581. 10.1093/eurpub/cks136 23042230

[65] Jagger C, Matthews RJ, Matthews FE, Spiers NA, Nickson J, Paykel ES, et al Cohort differences in disease and disability in the young-old: findings from the MRC Cognitive Function and Ageing Study (MRC-CFAS). BMC Public Health. 2007;7: 156 10.1186/1471-2458-7-156 17629910 PMC1947964

[66] Seeman TE, Merkin SS, Crimmins EM, Karlamangla AS. Disability trends among older Americans: National Health And Nutrition Examination Surveys, 1988–1994 and 1999–2004. Am J Public Health. 2010;100: 100–107. 10.2105/AJPH.2008.157388 19910350 PMC2791257

[67] Martin LG, Schoeni RF. Trends in disability and related chronic conditions among the forty-and-over population: 1997–2010. Disabil Health J. 2014;7: S4–S14. 10.1016/j.dhjo.2013.06.007 24456683 PMC4151570

[68] Schoeni RF, Martin LG, Andreski PM, Freedman VA. Persistent and growing socioeconomic disparities in disability among the elderly: 1982–2002. Am J Public Health. 2005;95: 2065–2070. 10.2105/AJPH.2004.048744 16254235 PMC1449484

[69] Martin LG, Schoeni RF, Andreski PM, Jagger C. Trends and inequalities in late-life health and functioning in England. J Epidemiol Community Health. 2012;66: 874–880. 10.1136/jech-2011-200251 22147749

[70] Doblhammer G, Fink A, Zylla S, Fritze T, Willekens F. Short-term trends in German Dementia prevalence, incidence, and mortality. Alzheimers Dement J Alzheimers Assoc. 2014;10: P279 10.1016/j.jalz.2014.04.527

[71] Bilotta C, Bergamaschini L, Nicolini P, Casè A, Pina G, Rossi SV, et al Frailty syndrome diagnosed according to the Study of Osteoporotic Fractures criteria and mortality in older outpatients suffering from Alzheimer’s disease: a one-year prospective cohort study. Aging Ment Health. 2012;16: 273–280. 10.1080/13607863.2011.609534 21995585

[72] Ratey JJ, Loehr JE. The positive impact of physical activity on cognition during adulthood: a review of underlying mechanisms, evidence and recommendations. Rev Neurosci. 2011;22: 171–185. 10.1515/RNS.2011.017 21417955

