# Supplementary material for: Population Aging at Cross-Roads: Diverging Secular Trends in Average Cognitive Functioning and Physical Health in the Older Population of Germany
Source: PLoS One. 2015 Aug 31;10(8):e0136583. doi: 10.1371/journal.pone.0136583 (PMC4556449; doi:10.1371/journal.pone.0136583)
Supplement: S1 File — (DOCX) [file pone.0136583.s002.docx]

**S1 File. Description of SF-12 Measures Included in the SOEP.**

The SF-12 module in the SOEP is modelled on the classic SF-12v2 Health Survey that uses 12 questions to measure functional health and well-being. It covers eight health domains and is measured based on the following questions:

1. ***Physical functioning*** *(PF): (1) When you ascend stairs, i.e. go up several floors on foot: Does your state of health affect you greatly, slightly or not at all? (2) And what about having to cope with other tiring everyday tasks, i.e. where one has to lift something heavy or where one requires agility: Does your state of health affect you greatly, slightly or not at all?*
2. ***Role limitations due to physical health problems*** *(RP): (1) Please think about the last four weeks. How often did it occur within this period of time that due to physical health problems you achieved less than you wanted to at work or in everyday tasks? (2)…that due to physical health problems you were limited in some form at work or in everyday tasks? [always, often, sometimes, seldom, never]*
3. ***Bodily pain*** *(BP): Please think about the last four weeks. How often did it occur within this period of time that you had strong physical pains? [always, often, sometimes, seldom, never]*
4. ***General health perceptions*** *(GH): How would you describe your current health? (very good, good, satisfactory, poor, bad).*
5. ***Energy and vitality*** *(VT): Please think about the last four weeks. How often did it occur within this period of time that that you used up a lot of energy? [always, often, sometimes, seldom, never]*
6. ***Social functioning*** *(SF): How often did it occur within this period of time that due to physical or mental health problems you were limited socially, i.e. in contact with friends, acquaintances or relatives? [always, often, sometimes, seldom, never]*
7. ***Role limitations due to mental health or emotional problems*** *(RE): (1) How often did it occur within this period of time that due to mental health or emotional problems you achieved less than you wanted to at work or in everyday tasks? (2)…that due to mental health or emotional problems you carried out your work or everyday tasks less thoroughly than usual? [always, often, sometimes, seldom, never]*
8. ***Mental health*** *(MH)*: *(1) How often did it occur within this period of time that you felt run-down and melancholy? (2)…that you felt relaxed and well-balanced? [always, often, sometimes, seldom, never]*
