# Supplementary material for: Population Aging at Cross-Roads: Diverging Secular Trends in Average Cognitive Functioning and Physical Health in the Older Population of Germany
Source: PLoS One. 2015 Aug 31;10(8):e0136583. doi: 10.1371/journal.pone.0136583 (PMC4556449; doi:10.1371/journal.pone.0136583)
Supplement: S2 File — (DOCX) [file pone.0136583.s003.docx]

**S2 File. Sensitivity Analyses Including Those Giving Oral Responses in 2006.**

The Symbol-Digit Test (SDT) in the SOEP is in principle designed for self-administration. Only in 2006 and only in exceptional circumstances, respondents could ask the interviewer to enter the digits they suggested (in 2012 oral response was not foreseen as an option anymore). The oral response mode was chosen by 17% of respondents to the 2006 cognitive module. Although the oral mode was more likely to be chosen by older and less educated persons (who tend to score lower on the SDT), they benefited from the interviewer assistance and attained higher average scores than those not opting-out from self-completion. Test scores of oral responders are thus not directly comparable to those of the majority of test participants.

The findings presented in the study (see Tables 4 and 5) are based on a sample of analysis that excludes those who opted out of self-completion and gave oral responses in 2006. This is justified by the fact that those not able to self-complete the test on a laptop (e.g., due to an impaired motor function) are also excluded from cognitive test participation in 2012 (when oral response was not an option). The rationale is thus a *consistent* exclusion of this population group in both survey waves. To test how the exclusion of those giving oral responses in 2006 in the sample of analysis may affect our results, we run sensitivity analyses using two strategies, (1) excluding test participants giving oral responses (results presented in the main text of the article) and (2) including test participants giving oral responses by way of imputing the score they would have obtained in the self-completion mode (results presented below).

We calculated predicted SDT scores for oral responders using OLS models that regress SDT scores on age, education, general health, work status, partnership status, the presence of a personal computer and a mobile phone in the household, body height, and scores on the second cognitive test administered in the SOEP in 2006, i.e., the animal naming test [for information about this test, see S1,S2]. These regressions attain an R-squared of about 0.3 (see Table A). The SDT variable we use for strategy 2 combines the raw scores for the majority sample with the predicted scores for those seeking interview help (see Fig.1 for sample sizes).

Analyses that include oral responders by way of imputing their test scores (Table B) yield estimates that point to more favorable trends in terms of cognition and less strongly negative trends in terms of physical and mental health compared to the results shown in Table 5. The exclusion of oral responders may thus imply an underestimation of the Flynn effect and an overestimation of the magnitude of physical health declines. The true population health trends will lie in-between the two sets of estimates based on the two strategies. We argue that oral responders are to be excluded from the 2006 sample, because those physically or cognitively *unable* to self-complete the SDT are also excluded in 2012. At the same time, it may be argued that selection of the oral mode was not restricted to those *not able* to self-complete the test; it may also have involved some that would have been able to self-complete the test in the absence of an oral response option. In this scenario, the exclusion of oral responders from the 2006 sample would result in an upward biased estimate of population health in 2006 and subsequently a downward biased estimate of health trends between 2006 and 2012. In any case, the general pattern of results remains the same irrespective of whether or not those giving oral responses are included in the sample of analysis (see Table B).

**References**

S1. Lang F, Weiss D, Stocker A, von Rosenbladt B. Assessing cognitive capacities in computer-assisted survey research: two ultra-short tests of intellectual ability in the German Socio-Economic Panel (SOEP). Schmollers Jahrb. 2007;127: 183–192.

S2. Schupp J, Herrmann S, Jaensch P, Lang FR. Erfassung kognitiver Leistungspotentiale Erwachsener im Sozio-oekonomischen Panel (SOEP) [Internet]. DIW Berlin; 2008. Available: http://www.diw.de/documents/publikationen/73/diw_01.c.85173.de/diw_datadoc_2008-032.pdf

**Table A. Regressions Based on which SDT-Scores for Oral Respondents in 2006 are Predicted.**

|  | Men | Women |
| --- | --- | --- |
| Age | -0.135*** | -0.174*** |
| Education (ref: ISCED 1) |  |  |
| ISCED 2 | 8.183* | 5.229* |
| ISCED 3 | 7.885* | 5.813** |
| ISCED 4 | 7.153 | 7.485** |
| ISCED 5 | 7.123 | 5.817* |
| ISCED 6 | 8.514* | 5.036* |
| ANT (animal naming task) | 0.284*** | 0.330*** |
| General health (GH) in 2006 | 0.042 | 0.052* |
| In paid work | 0.858 | 1.164 |
| Mobile phone in household | 1.285 | 0.824 |
| PC in household | 1.587* | 1.191* |
| Body height in cm | 0.085* | -0.004 |
| Presence of partner | 0.022 | -0.217 |
| Constant | -2.514 | 16.911* |
| Observations | 928 | 995 |
| R-squared | 0.268 | 0.302 |

*Sample:* All self-completers of the SDT test in 2006, aged 50-90. Based on this analysis, out of sample predictions are made for those using interviewer help in 2006. **** p<0.001, ** p<0.01, * p<0.05.*

**Table B. Sensitivity Analyses: Time Effects for Population Subgroups (Extended Sample).**

| *Men* | *SDT* | *PCS* | *MCS* | *PF* | *RP* | *BP* | *GH* | *VT* | *SF* | *RE* | *MH* | N |
| --- | --- | --- | --- | --- | --- | --- | --- | --- | --- | --- | --- | --- |
| Age 50-90 | 2.860*** | -0.634 | -1.237*** | -0.939* | -1.680*** | -0.831* | -0.125 | -0.244 | -1.360*** | -1.046** | -1.358*** | 2,642 |
|  |  |  |  |  |  |  |  |  |  |  |  |  |
| Age 50-64 | 3.039*** | -1.429** | -1.736** | -1.875*** | -2.308*** | -1.196* | -0.805 | -1.311* | -1.627** | -1.277* | -1.426** | 1,327 |
| Age 65-74 | 2.392*** | -0.361 | -1.163 | -0.721 | -1.633* | -0.933 | 0.522 | 0.449 | -1.293* | -0.990 | -1.438* | 887 |
| Age 75-90 | 3.252*** | 1.730 | -0.362 | 1.646 | 0.786 | 1.049 | 1.337 | 2.102* | -0.259 | 0.053 | -0.470 | 428 |
|  |  |  |  |  |  |  |  |  |  |  |  |  |
| Low education | 3.109*** | -1.039 | -0.733 | -1.130 | -1.857** | -1.281* | -0.500 | 0.842 | -0.894 | -0.583 | -1.827*** | 1,361 |
| Intermediate edu | 2.278*** | -0.621 | -1.722* | -1.101 | -1.877* | -0.649 | 0.003 | -1.456 | -2.075** | -1.571* | -0.815 | 594 |
| High education | 2.804*** | -0.083 | -1.948** | -0.727 | -1.507* | -0.311 | 0.359 | -1.391* | -1.965** | -1.572** | -1.063 | 720 |
|  |  |  |  |  |  |  |  |  |  |  |  |  |
| Age 50-64, low edu | 3.276*** | -2.778*** | -1.090 | -3.015*** | -3.657*** | -2.349** | -1.686* | -0.555 | -1.196 | -1.474 | -2.287** | 603 |
| Age 50-64, med/hi edu | 2.853*** | -0.449 | -1.762* | -0.996 | -1.402* | -0.325 | -0.255 | -2.002** | -2.175*** | -1.185 | -0.760 | 739 |
| *Women* | ***SDT*** | ***PCS*** | ***MCS*** | ***PF*** | ***RP*** | ***BP*** | ***GH*** | ***VT*** | ***SF*** | ***RE*** | ***MH*** | N |
| Age 50-90 | 2.416*** | -0.332 | -0.683 | -0.584 | -1.293** | -0.369 | 0.140 | 0.619 | -0.925* | -0.562 | -1.011** | 2,894 |
|  |  |  |  |  |  |  |  |  |  |  |  |  |
| Age 50-64 | 1.994*** | -1.256* | -1.446** | -1.651** | -2.330*** | -1.255* | -0.591 | -0.860 | -1.570** | -1.222* | -1.688** | 1,510 |
| Age 65-74 | 2.825*** | 1.468* | 0.698 | 1.467* | 0.820 | 0.938 | 1.914** | 2.916*** | 0.452 | 0.593 | 0.390 | 908 |
| Age 75-90 | 3.077*** | -0.247 | -0.757 | -0.377 | -1.359 | 0.091 | -0.528 | 1.196 | -0.959 | -0.405 | -1.524 | 476 |
|  |  |  |  |  |  |  |  |  |  |  |  |  |
| Low education | 2.358*** | 0.036 | -0.555 | -0.448 | -0.987 | -0.079 | 0.541 | 1.267* | -0.661 | -0.146 | -1.376** | 1,569 |
| Intermediate edu | 1.918*** | -0.997 | -0.998 | -1.166 | -1.654* | -0.740 | -0.781 | -0.791 | -1.280 | -0.900 | -0.949 | 905 |
| High education | 3.056*** | -1.085 | -0.721 | -0.422 | -2.413** | -1.409 | -0.262 | 0.610 | -1.244 | -1.673* | -0.296 | 445 |
|  |  |  |  |  |  |  |  |  |  |  |  |  |
| Age 50-64, low edu | 1.484* | -1.190 | -1.771* | -1.848* | -2.697** | -1.206 | -0.388 | -0.821 | -1.989* | -0.845 | -2.427** | 654 |
| Age 50-64, med/hi edu | 2.641*** | 0.911 | -0.056 | 0.465 | -0.220 | 0.625 | 1.650* | 2.083** | -0.249 | 0.072 | -0.429 | 801 |

*Sample*: First-time participants in cognitive testing in the SOEP in 2006 or 2012 (including those giving oral responses to the SDT in 2006). Population aged 50-90 at the time of interview. Regression analyses run for separate population groups and outcome measures; coefficients show time effects (2012 vs. 2006), controlling for age, age squared, and years of education.
