# Supplementary material for: Population Aging at Cross-Roads: Diverging Secular Trends in Average Cognitive Functioning and Physical Health in the Older Population of Germany
Source: PLoS One. 2015 Aug 31;10(8):e0136583. doi: 10.1371/journal.pone.0136583 (PMC4556449; doi:10.1371/journal.pone.0136583)
Supplement: S1 Table — Sample: First-time participants in cognitive testing in the SOEP in 2006 or 2012. Population aged 50–90 at the time of interview. Regression analyses run for separate population groups and outcome measures; coefficients show time effects (2012 vs. 2006), controlling for age, age squared, and years of education. Abbreviations: PF–physical functioning; RP–role physical; BP–bodily pain; GH–general health; VT–vitality; SF–social functioning; RE–role emotional; MH–mental health (for details on survey questionnaire wording, see S1 File). (DOCX) [file pone.0136583.s004.docx]

**S1 Table. Time Effects for Population Subgroups, SF-12 Sub-Dimensions.**

| *Men* | *PF* | *RP* | *BP* | *GH* | *VT* | *SF* | *RE* | *MH* | N |
| --- | --- | --- | --- | --- | --- | --- | --- | --- | --- |
| Age 50-90 | -1.643*** | -2.142*** | -1.418*** | -0.563 | -0.616 | -1.682*** | -1.411*** | -1.659*** | 2,345 |
|  |  |  |  |  |  |  |  |  |  |
| Age 50-64 | -2.480*** | -2.863*** | -2.010*** | -1.410** | -1.866*** | -1.887*** | -1.592** | -1.961*** | 1,206 |
| Age 65-74 | -1.330 | -1.899* | -0.882 | 0.312 | 0.568 | -1.451* | -1.266 | -1.306 | 779 |
| Age 75-90 | 1.069 | 0.661 | 0.119 | 1.296 | 1.810 | -0.950 | -0.468 | -0.684 | 360 |
|  |  |  |  |  |  |  |  |  |  |
| Low education | -2.220*** | -2.392*** | -2.176*** | -1.141* | 0.399 | -1.419* | -1.258* | -2.458*** | 1,159 |
| Intermediate edu | -1.934* | -2.482** | -1.358 | -0.609 | -1.878* | -2.243** | -1.875* | -1.023 | 551 |
| High education | -0.664 | -1.617* | -0.290 | 0.388 | -1.342 | -1.885** | -1.279* | -1.037 | 662 |
|  |  |  |  |  |  |  |  |  |  |
| Age 50-64, low edu | -3.922*** | -4.498*** | -3.702*** | -2.653** | -1.289 | -1.745* | -2.201** | -3.470*** | 528 |
| Age 50-64, med/hi edu | -1.455* | -1.812** | -0.831 | -0.675 | -2.306** | -2.211*** | -1.278 | -0.883 | 689 |
| *Women* | ***PF*** | ***RP*** | ***BP*** | ***GH*** | ***VT*** | ***SF*** | ***RE*** | ***MH*** | N |
| Age 50-90 | -0.870* | -1.358** | -0.406 | -0.100 | 0.296 | -0.805 | -0.546 | -1.317** | 2,506 |
|  |  |  |  |  |  |  |  |  |  |
| Age 50-64 | -1.867*** | -2.487*** | -1.402* | -0.876 | -0.800 | -1.604** | -1.227* | -1.776** | 1,389 |
| Age 65-74 | 1.029 | 0.851 | 1.452 | 1.870* | 2.342** | 1.027 | 1.050 | 0.017 | 761 |
| Age 75-90 | -0.087 | -0.912 | -0.124 | -0.704 | 0.892 | -0.919 | -1.080 | -2.232* | 356 |
|  |  |  |  |  |  |  |  |  |  |
| Low education | -0.464 | -0.610 | 0.151 | 0.480 | 1.201* | -0.551 | -0.179 | -1.762** | 1,298 |
| Intermediate edu | -1.310 | -1.906* | -0.749 | -0.688 | -1.189 | -0.898 | -0.553 | -1.175 | 811 |
| High education | -1.736 | -3.355*** | -2.145* | -1.430 | -0.114 | -1.545 | -1.967* | -0.591 | 418 |
|  |  |  |  |  |  |  |  |  |  |
| Age 50-64, low edu | -1.951* | -2.543** | -1.263 | -0.626 | -0.740 | -2.052* | -0.983 | -2.625** | 581 |
| Age 50-64, med/hi edu | -1.938** | -2.605*** | -1.632* | -1.173 | -1.008 | -1.371* | -1.462* | -1.252 | 818 |

*Sample*: First-time participants in cognitive testing in the SOEP in 2006 or 2012. Population aged 50-90 at the time of interview. Regression analyses run for separate population groups and outcome measures; coefficients show time effects (2012 vs. 2006), controlling for age, age squared, and years of education. *Abbreviations*: PF – physical functioning; RP – role physical; BP – bodily pain; GH – general health; VT – vitality; SF – social functioning; RE – role emotional; MH – mental health (for details on survey questionnaire wording, see S1 File).
